# The sympathetic nervous system response to a Continuous Performance Task

**DOI:** 10.17912/micropub.biology.001059

**Published:** 2024-06-12

**Authors:** Brandon R. Bautista, Jessica Gurning, Megan Marks, David Ortyn, Rankin Salinas, Lisa E. Olson

**Affiliations:** 1 Biology, University of Redlands, Redlands, California, United States

## Abstract

A Continuous Performance Task is an example of a mental stressor which requires vigilance, attention, and effort. We hypothesized that a sympathetic nervous system response would be evident from a resting baseline period to this attention test, and explored if physiological measures were correlated to state and trait anxiety, perceived stress, mindfulness, and performance on the task. In 20 undergraduates, blood pressure and skin conductance increased due to the attention test but heart rate variability did not change. The physiological variables did not correlate to psychological variables; there was a trend of higher perceived stress correlating to lower foil accuracy rate ( *p* = 0.09). ClinicalTrials.gov ID: NCT06098352

**Figure 1.**
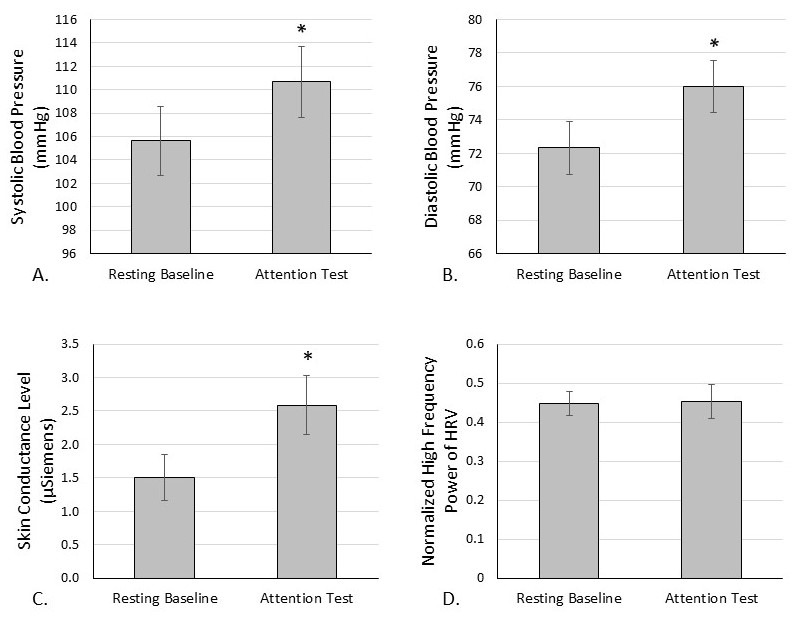
The sympathetic nervous system response to an attention test. The PEBL Continuous Performance Task requires vigilance and attention from the participant to respond correctly to presented stimuli. Systolic (A) and diastolic (B) blood pressure and skin conductance level (C) were elevated. Normalized high frequency power (D), a measure of parasympathetic tone, remained the same. * = *p* < 0.001

## Description

The sympathetic nervous system or “flight or flight response” can be triggered by physical, emotional, or mental stressors and can be influenced by psychological traits including mindfulness fostered by meditation (Chida & Hamer, 2008; Pascoe et al., 2021) . Sympathetic activation manifests in multiple systems, including various neurohormonal, cardiovascular, respiratory, digestive, motor, and electrodermal effects (Bali & Jaggi, 2015; Wehrwein et al., 2016) . Many of these responses are countered by the parasympathetic nervous system. In this study, 20 healthy undergraduate students were administered the Psychology Experiment Building Language (PEBL) Continuous Performance Task requiring vigilance and attention. The data on mindfulness and attention is conflicting (Prakash et al., 2020) . We hypothesized that this task would induce sympathetic activation, that the magnitude of activation would be negatively correlated to mindfulness, and that the performance on the task would be positively correlated to mindfulness.

The PEBL Continuous Performance Task attention test caused a sympathetic nervous system response as evidenced by elevations in systolic blood pressure (Cohen’s *d* = -1.035; *p* < 0.001), diastolic blood pressure (Cohen’s *d* = -1.171; *p* < 0.001), and skin conductance level (Cohen’s *d* = -1.194; *p* < 0.001) compared to baseline ( Figure 1 ). High frequency power of HRV, often considered reflective of parasympathetic tone, was not decreased however (Cohen’s *d* = -.035; *p* > 0.05). This is similar to studies in which high frequency power of HRV was not decreased due to a psychosocial stressor (Grimley et al., 2019) or an anger recall task (Whinery et al., 2022) and adds to speculation about the validity of this measure of vagal tone (Thomas et al., 2019) .

To explore whether the level of sympathetic nervous system activation was related to psychological traits, we also asked participants to complete questionnaires measuring state and trait anxiety, perceived stress, and mindfulness. The psychological variables were correlated to each other in directions that were expected. Mindfulness was negatively correlated with state anxiety ( *r* = -0.81), trait anxiety ( *r* = -0.81), and perceived stress ( *r* = -0.67; *p* ’s < 0.002). State anxiety, trait anxiety, and perceived stress were all positively correlated with each other ( *r* ’s 0.83 – 0.92; *p* ’s < 0.001). Baseline physiological variables did not correlate to psychological variables ( *p* ’s > 0.05), but there was a trend of higher perceived stress correlating to lower foil accuracy rate on the PEBL Continuous Performance Task ( *r* = -0.54; adjusted Benjamini-Hochberg *p* = 0.09). The magnitude of change in the physiological variables from baseline to the attention test did not correlate to any psychological variables ( *p* ’s > 0.05). However, our sample size was limited and thus we did not have statistical power to detect small correlations. Replication in another population would be helpful to confirm our results and allow more generalizability.

## Methods

The study was prospectively approved by our Institutional Review Board (approval number 2013-31-REDLANDS) and retrospectively registered with clinicaltrials.gov (NCT06098352). Data collection occurred in September 2013. Participants provided informed consent, and the experiment was performed according to the Declaration of Helsinki. Participants were first year, first semester undergraduate students at a small liberal arts university in California, USA who were enrolled in a Students Together Empowering Peers course. All students in this course were originally invited to participate in a larger study on the impact of a meditation intervention, but that trial was cancelled due to low recruitment. Data from the baseline measurements are presented here for the participants who did enroll. Exclusion criteria included age under 18 years, severe mental health issues, current use of anti-anxiety medication, or lack of English language proficiency. Participants were compensated $10 for the study.

The 20 participants included 5 males, 15 females, and 1 undisclosed; all were ages 18-19. The majority were non-White: 10 Hispanic/Latino, 1 African-American, 1 Korean, 1 Romanian, 5 White, and 2 undisclosed. None had been diagnosed with Attention Deficit Disorder.

Participants completed pen-and-paper versions of the State Trait Anxiety Inventory (Spielberger et al., 1970) , the Perceived Stress Scale-10 (Cohen et al., 1983) , and the Mindful Awareness and Attention Scale (Brown & Ryan, 2003) . Physiological measures were then collected for an 8 minute baseline period while listening to recorded beach sounds, and then during the 14 minute PEBL Continuous Performance Task (Mueller & Piper, 2014) . This vigilance/attention test is a free, open-source ( http://pebl.sourceforge.net ) version of the Conner’s Continuous Performance Task (Homack & Riccio, 2006) in which participants are instructed to press the spacebar when any letter except “X” appears. The foil accuracy rate is number of trials where the participant correctly did not press spacebar divided by the number of trials with the letter “X” (the foil), with higher values indicating better attention.

Physiological measures were captured with the same equipment and software as previously described (Whinery et al., 2022) . Heart Rate Variability (HRV) and skin conductance level were captured continuously. Normalized high-frequency (0.15 Hz–0.4 Hz) power of HRV was calculated as high frequency power/total power. Blood pressure was taken at the end of the baseline period, then 2 minutes into the attention task, and 10 minutes into the attention task. Values at 2 minutes and 10 minutes were not statistically different, and data presented here are from the 2 minute time point.

Data were analyzed using SPSS statistical software (International Business Machines Corporation, Armonk, NY) with an alpha of 0.05. Assumptions of all tests were checked, including the Shapiro-Wilk test for normality. Data were normally distributed without outliers. Physiological variables were compared from baseline to attention test periods using paired samples t-tests. Correlations were tested using Pearson's product-moment correlation. The Benjamini-Hochberg correction for multiple testing was used to control the false discovery rate to 0.05 (McDonald, 2014) .
